# Disease evolution and organ damage accrual in patients with stable UCTD: a long-term monocentric inception cohort

**DOI:** 10.1136/rmdopen-2023-003967

**Published:** 2024-04-25

**Authors:** Chiara Tani, Francesca Trentin, Alice Parma, Dina Zucchi, Chiara Cardelli, Chiara Stagnaro, Elena Elefante, Viola Signorini, Linda Carli, Maria Laura Manca, Marta Mosca

**Affiliations:** 1 Department of Clinical and Experimental Medicine, University of Pisa, Pisa, Italy; 2 Rheumatology Unit, Department of Clinical and Experimental Medicine, Pisa, Italy; 3 Rheumatology Unit, University of Pisa, Pisa, Italy; 4 Rheumatology Unit, Department of Clinical and Experimental Medicine, University of Pisa, Azienda Ospedaliero Universitaria Pisana, Pisa, Italy; 5 Department of Medical Biotechnologies, University of Siena, Siena, Italy; 6 University of Siena, Siena, Italy; 7 Clinical and Experimental Medicine, Rheumatology Unit, Pisa, Italy; 8 Department of Clinical and Experimental Medicine, Rheumatology Unit, Azienda Ospedaliero Universitaria Pisana, Pisa, Italy; 9 Rheumatology Unit, AOU Pisana, Pisa, Italy; 10 University of Pisa, Pisa, Italy

**Keywords:** Connective Tissue Diseases, Autoimmune Diseases, Glucocorticoids

## Abstract

**Objectives:**

Undifferentiated connective tissue diseases (UCTDs) are systemic autoimmune conditions that cannot be diagnosed nor classified as defined CTD; the majority maintains an undifferentiated profile (stable UCTD, sUCTD) over time. Data on long-term outcomes of sUCTD are lacking.

**Methods:**

Retrospective longitudinal analysis of an inception cohort of 141 patients with sUCTD.

Disease evolution and damage accrual were evaluated at 1, 5 and 10 years. Partial least square (PLS) regression was used to identify the basal variables contributing to damage accrual at 1, 5 and 10 years of follow-up. Trend of damage over time was compared with a cohort of age-matched and sex-matched patients with systemic lupus erythematosus (SLE) by means of Nelson-Aalen analysis.

**Results:**

11.3% of patients evolved to a definite CTD after a median 11 years (IQR 6–25) from the first symptom. At last visit, 10% were on glucocorticoids and 6% on immunosuppressive therapy. In 27.3%, at least one item of organ damage was recorded according to the SLICC/DI score (mean score 1.19±0.46). At PLS analysis, age at diagnosis and age at first symptoms were related to damage at 1 year, not taking antimalarials and taking immunosuppressants were associated with damage at 5 years.

The mean survival without damage was 9.3 years in sUCTD and 8.4 years in SLE. The 10-year probability without damage was 62% and 23% in SLE and sUCTD, respectively (p=0.015).

**Conclusions:**

Although less significantly impacted than in patients with SLE, in the long-term UCTDs can accumulate organ damage and evolve into defined connective tissue diseases.

**WHAT IS ALREADY KNOWN ON THIS TOPIC**

- Undifferentiated connective tissue diseases (UCTDs) are systemic autoimmune conditions that cannot be diagnosed nor classified as defined CTD; the majority maintains an undifferentiated profile (stable UCTD, sUCTD) over time. Data on long-term outcomes of sUCTD are lacking.

**WHAT THIS STUDY ADDS**

- This is the first study assessing the disease burden in patients with UCTD, in terms of organ damage. According to our results, sUCTD represents a chronic condition that, despite its mild clinical presentation at onset and during the initial years of follow-up, over the years can be associated with organ damage accrual and late disease evolution.

**HOW THIS STUDY MIGHT AFFECT RESEARCH, PRACTICE OR POLICY**

- This study highlights the importance of careful disease monitoring, implementation of organ damage prevention strategies and effective management of comorbidities in patients with UCTD.

## Introduction

 ‘Undifferentiated connective tissue disease’ (UCTD) is a term that describes systemic autoimmune conditions that cannot be diagnosed nor classified as defined CTD.^1^,^4^

Literature data have shown that among UCTDs at onset up to 40% will evolve into defined CTDs, while the remaining will maintain an undifferentiated profile.^5 6^ These latter are the so-called ‘stable UCTD’ (sUCTD) a distinct clinical entity with peculiar clinical findings; the proposed preliminary classification criteria for sUCTD include at least one clinical manifestation of CTDs, positive antinuclear antibodies and disease duration of at least 3 years to distinguish these conditions from ‘evolving UCTD’.^4^

sUCTDs are the focus of this work. As of this writing, many previous studies have focused on the risk of progression to CTDs and investigated clinical features or biomarkers that could help predict which patients are more likely to develop new clinical and immunological features that could eventually lead to a formal classification of definite CTD diagnosis.^7^ However, data on long-term outcome and disease burden in patients with sUCTD remain scarce, and further research is needed to better understand these patients’ prognosis.^8^

## Methods

### Study design

This is a retrospective analysis of prospectively collected data from a monocentric, inception cohort of patients with sUCTD.

### Patients

Although there are no defined and validated classification criteria for UCTDs, we referred to the following preliminary criteria: (1) signs and symptoms suggestive of a CTD, but not fulfilling criteria for a defined CTD, (2) positive antinuclear antibodies on two separate measurements and (3) a disease duration of at least 3 years.^3^

Among those, patients regularly followed at our centre since diagnosis and with at least 1 year of follow-up were eligible for inclusion in the present analysis. Patients were included in the analysis if they had attended at least one visit every 2 years at our centre.

Patients with <1 year of follow-up, incomplete follow-up or incomplete clinical history, or lack of seroimmunological parameters were excluded.

ANA positivity was confirmed in all patients with an indirect immunofluorescence on HEp-2 cells, performed at our local laboratory.

For comparison, a control group of sex-matched and age-matched patients with systemic lupus erythematosus (SLE) fulfilling the 2019 American College of Rheumatology (ACR)/European Alliance of Associations for Rheumatology classification criteria was used.

### Data collection

The following demographic and clinical variables were collected: date of birth, age, gender, age at first symptom and at UCTD diagnosis, duration of the follow-up since diagnosis (defined as the interval between first visit and last observation at our centre), presenting symptoms and cumulative manifestations, serological profile, presence of comorbidities.

Therapies were also recorded as ‘initial therapies’ (prescribed at the time of the diagnosis), ‘cumulative therapies’ (at least once during the entire follow-up) and ‘therapies at last observation’ (ongoing therapies at last visit at our clinic). The following treatment categories were considered: glucocorticoids (GC), antimalarials (hydroxychloroquine or chloroquine, AM), traditional immunosuppressive drugs (IS, including azathioprine, methotrexate, mycophenolate mofetil, cyclosporine A, cyclophosphamide).

Organ damage was assessed using the SLICC/ACR Damage Index (SDI) and was calculated at 1, 5 and 10 years from disease onset. By definition, only events that occurred after disease onset were recorded. Damage was then analysed as a continuous (total damage score) and as a dichotomous variable (presence or absence of damage) at each predefined time point.^9^

Based on the items of the SDI, we focused our analysis on categories of damage: (1) disease related damage (ie, renal damage, cognitive impairment, Jaccoud’s arthropathy, alopecia, splenectomy) (2) drug toxicity (ie, osteoporosis, diabetes, cataracts, avascular necrosis, premature ovarian failure), (3) cancer (excluding dysplasia) and (4) other types of organ damage (ie, cardiovascular).

Age and disease duration at first damage item were also recorded.

### Statistical analysis

Statistical analysis was performed by using R and Stata packages for MacOs X.

Continuous variables were presented as mean and SD; variables with a skewed distribution (by the Shapiro-Wilk test) were given as median (IQR).

Group values were compared by Student’s t-test or Mann-Whitney test, if appropriate, while proportions by the χ^2^ test. In the UCTD cohort, partial least square (PLS) regression was used to identify the basal variables maximally contributing to damage accrual after 1 year.

Variables with a Variable Importance in Projection (VIP) score >2.50 (a measure of variable relevance in the model) were considered significant for the association with the damage accrual. The analysis was repeated at 5-year and 10-year follow-up.

We used the Nelson-Aalen analysis, as an estimator of the cumulative hazard rate function of the sample, divided by patients with UCTD and SLE, at 10-year follow-up. Functions of groups were compared using log-rank test.

Finally, we estimated the effect of group (UCTD or SLE) on SLICC/DI at last visit, using logistic regression. We used the R package MatchIt to estimate the effect of the group on damage while controlling for the following covariates: sex, age at last visit, age at onset and disease duration. In this analysis, we have chosen the optimal full matching method, while the propensity score was estimated with probit regression. Then, we fit logistic regression model with SLICC/ID as the outcome and the group, covariates and their interaction as predictors, and included the full matching weights in the estimation.

All the tests above were two tailed, and p values <0.05 were considered significant. When necessary, a multiple testing correction was applied.

## Results

Two hundred and fifty-six clinical records were reviewed, and 141 patients were eligible for this analysis, 4 males (2.8%) and 137 females (97.2%), all patients were Caucasian. The epidemiological characteristics of the whole cohort are summarised in table 1. The most frequent presenting symptoms were arthralgias/arthritis, Raynaud’s phenomenon and skin rash reported in 51.7%, 49.6% and 17.7% of patients, respectively. These manifestations were also the most prevalent over time (table 1).

**Table 1:** Clinical-demographic data of patients with sUCTD in comparison with SLE-matched controls

Variable	UCTD(n=141)	SLE(n=153)	P value
Age at last visit	48.5±12.6	45.6±12.2	0.022
Follow-up duration (first visit-last visit, years)	8.0 (5–13)	7.0 (4–14)	NS
Age at first symptom (years)	33.5 (25–44)	32.0 (24–42)	NS
Age at diagnosis (years)	37.0 (29–47)	36.0 (28–46)	NS
Duration of symptoms at first visit (months)	12.0 (6–36)	NA	
Disease duration at last visit (years)	8.0 (5–12)	9.0 (3–15)	NS

Data are expressed as median and intequartile range IQR.

NA, not availableNS, not significantSLEsystemic lupus erythematosussUCTDstable UCTDUCTDundifferentiated connective tissue disease

As far as the serological profile is concerned, by definition all patients were ANA positive; anti-Ro and aPL (including lupus anticoagulant and/or anticardiolipin and/or anti beta2glycoprotein I) were the most prevalent autoantibodies observed in 27.3% and 24.8% of patients, respectively, while anti-U1RNP, anti-dsDNA and anti-La were observed in around 5% of the patients each (table 1). Hypocomplementaemia was also frequent, being recorded in 45.3% of patients.

Of note, 10/141 (7%) patients have both sicca symptoms and anti-Ro positivity; however, these patients had negative functional tests, negative salivary glands ultrasounds and/or negative salivary gland biopsy at baseline or during the follow-up.

**Figure 1:**
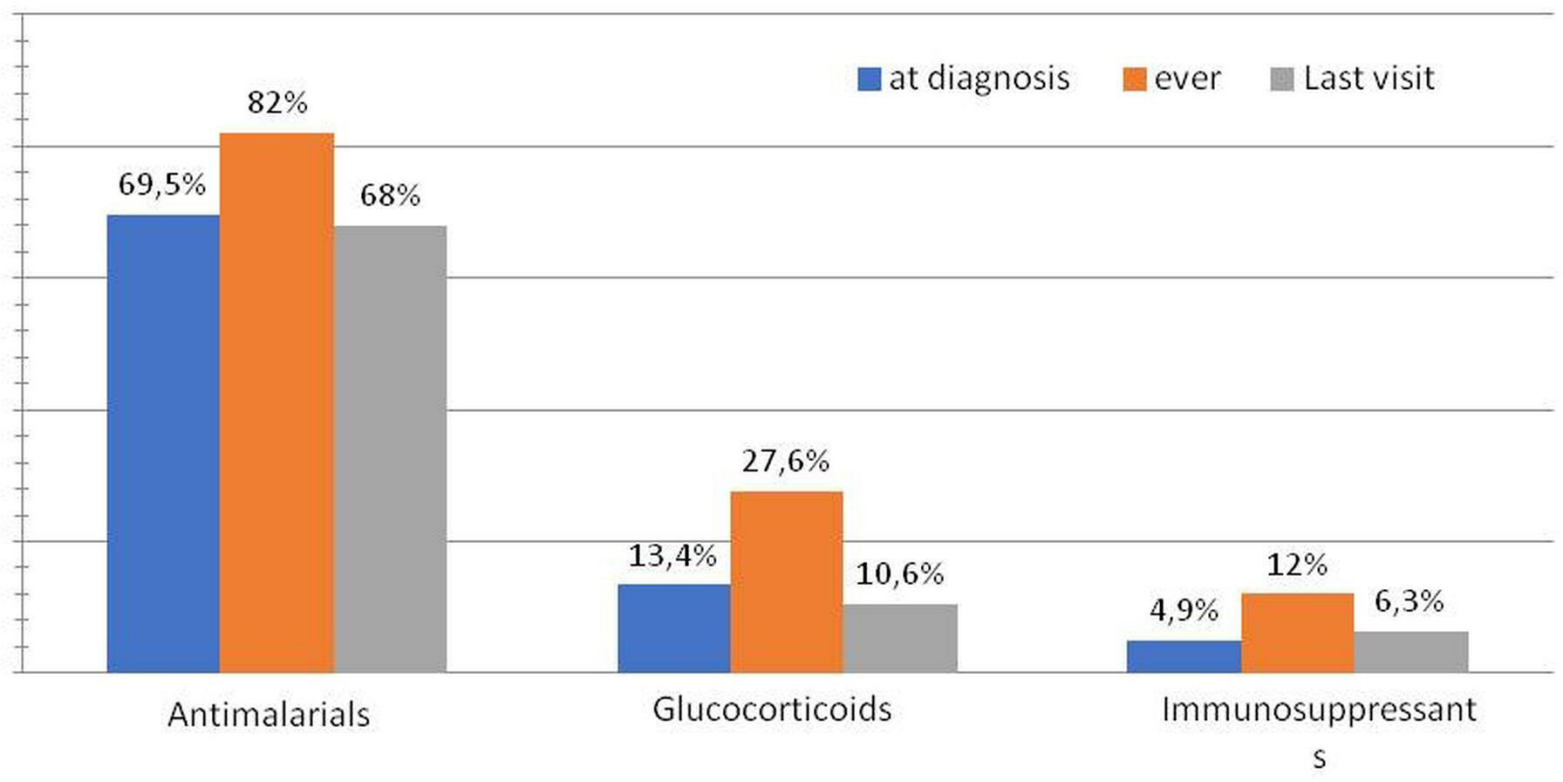
Therapies prescribed at the time of diagnosis, during the follow-up and at last visit (%).

At least one comorbidity was present in the majority of the patients (108, 76%) being autoimmune thyroiditis and fibromyalgia the most common (in 29% and 26% of the patients, respectively). Interestingly, overall, organ-specific autoimmune conditions were present in 53 (37.5%) of patients with sUCTD (table 1). Therapies received at diagnosis and over time are summarised in figure 1. At the time of diagnosis, the majority of patients received AM (69.5%) and/or GC (13.4%) while only a minority received IS (4.9%). During the entire follow-up, the treatment burden tends to increase for all these categories (81% AM, 27.6% GC, IS 12%). At last visit, 68%, 10% and 6.3% of the patients were still on AM, GC and IS, respectively. Interestingly, 14.8% of the whole cohort never received these therapies but only symptomatic therapies (NSAIDs, paracetamol).

### Disease evolution

Of note, 16 patients (11.3%) evolved in a definite CTD during the follow-up; the evolution occurred after a mean time of 14±9.8 years from the first symptom (median 11 years, IQR 6–25). Specifically, 10 patients (7%) evolved into SLE, 2 (1.4%) into Sjögren’s syndrome (SS), 1 (0.7%) into idiopathic inflammatory myopathy, 1 (0.7%) into systemic sclerosis and 2 into overlap syndrome (1 rheumatoid arthritis and SS, 1 IIM and SS).

Baseline serological data of patients who evolved into CTDs compared with patients who did not evolve are shown in online supplemental table S1. Briefly, the percentage of positivity at baseline of anti-dsDNA and anti-Ro was significantly higher in evolved patients than in non-evolved patients (18.7% vs 3.5%% p=0.04; 6.2% vs 4.3%, p=0.04); no statistical difference was found for the other antibody specificities.

### Damage accrual

At last observation, in 38 patients (27.3%) at least one item of organ damage was recorded according to the SLICC/DI score with a mean score of 1.19±0.46 (median, IQR 1–1); neoplasia was the most prevalent event (14 cases during the follow-up, 9.9%) while disease-related damage was reported in 6 cases (4.2%) and drug-related damage in only 2 cases (1.4%), other types of organ damage were present in 15 patients (online supplemental table S3).

Overall, the first damage occurred at a mean age of 44 years±12 (median 43, 38–54) and after a mean disease duration of 7.8 years±7.4 (median 7, 2–10). Interestingly, five patients (3.67%) already had organ damage after 1 year of follow-up.

Clinical characteristics of patients with UCTD with or without damage at last observation are reported in table 2. At univariate analysis, male gender resulted the only variable associated with damage at last visit (p=0.02).

**Table 2:** Clinical characteristics of patients with UCTD with or without damage at last observation

Variable	UCTD without damage at last visit	UCTD with damage at last visit	P value
Gender (female, %)	100/101	32/35	0.03
Age at diagnosis (median, IQR)	36.0 (18.0)	56.0 (14.0)	0.63
Age at first symptoms (median, IQR)	33.0 (18.0)	54.0 (10.0)	0.59
Disease duration at last visit (median IQR)	8 (6)	8 (7)	0.70
Therapy with antimalarial drugs (%), ever	86	76	0.25
Therapy with glucocorticoids (%), ever	25	40	0.70
Therapy with immunosuppressive drugs (%), ever	13	11	0.70

UCTDundifferentiated connective tissue disease

At PLS analysis, age at diagnosis (VIP=3.80) and age at first symptoms (VIP=3.57) were the variables more closely related to damage accrual at 1 year. At 5 years, damage was associated with not taking antimalarials (VIP=2.54) and with taking immunosuppressants (VIP=2.81). PLS analysis performed at 10-year follow-up did not identify any basal variables related to damage accrual.

The occurrence of organ damage in UCTD was compared with a control group of 153 patients with established SLE matched for age at diagnosis and disease duration; comparative demographic and clinical data between UCTD and SLE are summarised in table 3. A more detailed description of the control group is summarised in online supplemental table S2.

**Table 3:** sUCTD clinical data

UCTD	N=141
Presenting symptoms, no (%)	
Arthralgias/arthritis	73 (51.7)
Raynaud’s phenomenon	70 (49.6)
Skin rash (any type)	25 (17.7)
Constitutional	22 (15.6)
Haematologic	16 (11.35)
Sicca syndrome	15 (10.6)
Serositis	3 (2.1)
Cumulative disease manifestations, no (%)	
Articular	98 (69.5)
Raynaud’s phenomenon	94 (66.6)
Sicca syndrome	65 (46)
Constitutional	65 (46)
Skin rash (any type)	34 (24.1)
Haematologic	23 (16.3)
Serositis	5 (3.5)
Immunological characteristics, no/total number of test available (%)	
ANA+	141/141 (100)
Anti-Ro	36/132 (27.3)
aPL+, no (%)	35/141 (24.8)
Anti-RNP+, no (%)	7/127 (5.5)
Anti-dsDNA+	7/134 (5.22)
Anti-La	6/131 (4.5)
Anti-Sm+, no (%)	2/127 (1.6)
Direct Coomb’s test, no (%)	16/111 (14.4)
Hypocomplementaemia, no (%)	64/141 (45.3)
Comorbidities, no (%)	
None	33 (24)
Autoimmune thyroiditis	41 (29)
Fibromyalgia	38 (26)
Arterial hypertension	19 (13.4)
Steoporosis	15 (10.6)
Autoimmune gastritis	4 (2.8)
Coeliac disease	3 (2.1)
Psoriasis	3 (2.1)
Primary biliary cirrhosis	2 (1.4)

sUCTDstable UCTDUCTDundifferentiated connective tissue disease

As far as damage accrual is concerned, at univariate analysis, at last visit patients with UCTD had lower SLICC/DI scores with respect to SLE patients (0.31 vs 1.01, p<0.001); however, the proportion of patients with at least one item of SLICC/DI was not statistically different (27% in UCTS vs 36% in SLE, p=0.1).

Nelson-Aaron analysis comparing UCTD versus SLE was performed at 10 years of follow-up. Results of Nelson-Aaron function calculation were reported in online supplemental tables S4 and S5.

**Figure 2:**
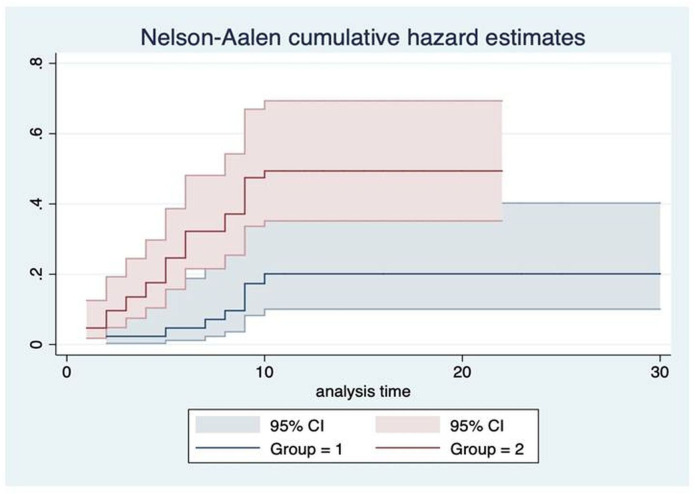
Nelson-Aalen cumulative hazard estimates. Group 1=UCTD; group 2=SLE. SLE, systemic lupus erythematosus; UCTD, undifferentiated connective tissue disease.

The Nelson-Aalen plot is displayed in figure 2 to show the cumulative hazard estimates of the two groups of subjects. Lastly, patients with SLE exhibited a percentage of events of 42% vs 22% in the UCTD group (log-rank test; p=0.011).

In the logistic regression, the effect of group on damage accrual was significant, also when we controlled for the covariates (p=0.010). In fact, patients with UCTD had lower SLICC/DI scores with respect to SLE subjects (0.31 vs 1.01). However, the percentage of patients with at least one item of SLICC/DI was not statistically different (27% in UTCD vs 36% in SLE, p=0.1).

## Discussion

In this study, we described the long-term outcomes of an inception cohort of patients with sUCTD; to the best of our knowledge, this is the first study focusing on damage accrual of sUCTD over a long-term follow-up period. Indeed, over the last years, the great effort of the scientific community has focused on the rate of evolution of UCTD to CTDs, on the identification of predictive factors and potential triggers for the evolution.^10 11^

As reported in previous studies,^5 6^ also in our inception cohort the clinical course of sUCTD was mild in the majority of the cases and the requirement of therapies was modest with 15% of patients never requiring a specific treatment other than symptomatic drugs. Interestingly, however, the amount of medication taken during follow-up has been increasing and even after a long-term follow-up the majority of patients were still on treatment, especially with antimalarials; moreover, at last visit roughly 10% were still on GC and 6% on immunosuppressive therapy. This is an interesting point if we parallel these data with data reported in SLE patients; indeed, in recent SLE cohorts, at last visit up to 50%–60% of SLE patients were still on GC and 70%–80% received immunosuppressive therapy.^12 13^ Thus, the treatment burden in sUCTD seems lower than SLE but not neglectable.

In this study, we also reported the evolution rate to definite CTD: 11.3% of sUCTD evolved into a classifiable CTD during the follow-up after a median of 11 years. The evolution rate observed in our patients is lower than reported in other cohorts in the literature; this is not surprising given that we included only patients with a disease duration of at least 3 years while the evolution is more likely in the first years after disease onset.^5^,^7^ Thus, our data demonstrated that sUCTD still is at risk of evolving into more complex diseases over time, meaning that a careful and long-term clinical monitoring is needed also in these mild conditions.

Another important aspect that emerged from this analysis is that the diagnostic delay can be very long in these patients; this could be explained by the fact that the initial symptoms of the disease can be mild and non-specific, leading the physician (usually the general practitioner) to consider other possible explanations beforehand as potential UCTD mimickers can include, for instance, infections, hypothyroidism, neoplasms.

Lastly, a high prevalence of comorbid organ-specific autoimmune diseases was reported during the entire follow-up, the most frequent being autoimmune thyroiditis in one-third of patients. This finding further reinforces the need for monitoring over time of the condition itself and any associated emerging comorbidities.

The innovative information provided by this study is the data on organ damage in sUCTD; at last observation, more than a quarter of patients presented at least one item of organ damage; only 4% of patients already accrued organ damage within the first year of the disease, while in the majority of patients, the first event was recorded after long disease duration. A control group of patients with SLE was used as reference for organ damage; as expected, over time SLE patients had a greater damage accrual than sUCTD, however, surprisingly, the percentage of patients with at least one item of organ damage was similar in the two cohorts after a similar follow-up. Indeed, in both cohorts, roughly one-third of patients was presenting a SLICC/DI>0 at last visit.

Thus, in this study clearly emerged that organ damage accrual can be an issue also in patients with UCTD, especially in the long-term management. Paralleling other diseases such as SLE, we know that organ damage is an important determinant of patients’ long-term outcome in terms of further damage, quality of life and mortality. Therefore, organ damage prevention should be a target also in the management of patients with UCTD.

Age at disease onset and at diagnosis resulted independent predictors associated with organ damage accrual in sUCTD; this is not surprising because these variables are clearly associated with organ damage also in SLE patients, but in SLE, other variables are much more important such as chronic GC therapy, persistent disease activity, occurrence of flares and some types of organ involvement (ie, renal) and comorbidities (ie, antiphospholipid syndrome).^14^,^16^ These factors are under-represented in patients with sUCTD or not present at all; thus, this could explain why these factors do not emerge as important contributors of the disease burden also in sUCTD.

On the other hand, in our cohort, treatment with antimalarial drugs resulted independently associated with less damage accrual at the 5 years’ time point. This is a very important finding that, if confirmed in larger studies, might have several clinical implications.

First of all, because there is no drug officially approved for the treatment of UCTD, these data can add an important piece of knowledge to support the use of these drugs in UCTD. Indeed, in the absence of rigorous prospective epidemiological data, it is still unclear if the use of drugs developed for other indications (such as antimalarials) may be useful for patients with UCTD.

The protective role of antimalarials against damage accrual is well established in SLE but some evidence of their usefulness also in UCTD has already been indirectly identified in animal models some years ago.^17^

In conclusion, these data highlight the fact that UCTD is chronic diseases that despite being clinically mild at onset and during the first years of follow-up can still have a long-term poor prognosis because of a disease progression, still possible after several years from disease onset, or because of the impact that the disease can have on the patient’s life because of irreversible organ damage.

The study has some limitations; the first point relies on the fact that the instrument we used to score organ damage was developed and validated in SLE cohorts while this is the first time that it was used in patients with UCTD. Indeed, there are no UCTD-specific tools to evaluate organ damage for UCTD; however, considering the clinical similarities between UCTD and SLE, we can assume that SLICC/DI is a reliable proxy outcome measures also in this cohort.

Moreover, the absence of definitive and validated classification criteria for sUCTD is certainly a cause of possible nosographic and diagnostic uncertainty. Furthermore, the presence of disease-specific autoantibodies (ie, anti-Sm, anti U1RNP, anti-dsDNA) can make the assessment even more difficult. In this context, the border between sUCTD and ‘early CTD’ can be very blurred.

On the other hand, in our opinion, the novelty of the data presented and the long-term follow-up of the patients starting from disease onset are the main strengths of this study.

In conclusion, this is the first study assessing the disease burden in patients with UCTD, in terms of organ damage. Further studies are ongoing to evaluate predictors and risk factors for long-term outcomes in larger cohorts of patients with sUCTD.

From the clinician’s perspective, these data highlight the importance of careful disease monitoring, organ damage prevention strategies and comorbidities management in patients with UCTD.

## supplementary material

10.1136/rmdopen-2023-003967 online supplemental file 1

## Data Availability

Data are available on reasonable request.
