# Supplementary material for: Disease evolution and organ damage accrual in patients with stable UCTD: a long-term monocentric inception cohort
Source: RMD Open. 2024 Apr 25;10(2):e003967. doi: 10.1136/rmdopen-2023-003967 (PMC11057319; doi:10.1136/rmdopen-2023-003967)
Supplement: online supplemental file 1 [file rmdopen-10-2-s001.pdf]

Supplementary files

Table S1: serological profile at baseline evaluation of UCTD patients who evolved to CTDs versus not evolved

	Evolved to CTDs	Not evolved to CTDs	P *
Anti-dsDNA %	18.7%	3.5%	**0.04**
Anti-Ro %	6.25%	4.3%	**0.04**
Anti-La %	6.25%	4.5%	Ns
aPL %	37.5%	23.2%	Ns
Anti RNP %	6.25%	0%	Ns
Anti- Sm	1.8%	0%	Ns

+ Fisher Exact test

Ns: not significant ($p>0.05$)

Table S2: disease characteristics of SLE (N=153) patients used as control group

**clinical characteristics**	**% of patients**
Joint involvement (ever)	68.6%
Muco-cutaneous involvement (ever)	55%
Hematological involvement (ever)	52.7%
Renal involvement (ever)	43.8%
Cardio-pulmonary involvement (ever)	19.4%
Neuropsychiatric involvement (ever)	11.7%
Patients with active disease (SLEDAI >4) at last visit	15.8%
Median SLEDAI among active patients	6 (IQR 6-9)
Patients with SDI >0 at last visit	49.6%
Median SDI (among patients with SDI>0)	2 (IQR 1-3)
Patients with fibromyalgia	10.8%
Patients with concomitant thyroid disease	24.3%

Table S3: type of damage at last evaluation

SLICC-DI Items	Number of patients	
Malignancy	14	3 breast cancer<br>2 non-Hodgkin lymphoma<br>2 thyroid cancer<br>2 non-melanoma skin cancer<br>1 ovarian cancer<br>1 Hodgkin lymphoma<br>1 chronic myeloid leukemia<br>1 myelodysplastic syndrome<br>1 systemic mastocytosis
Ocular damage	6	1 cataract<br>5 retinal changes
Neuropsychiatric damage	6	3 cognitive impairments,<br>1 seizures,<br>2 peripheral neuropathies
Musculoskeletal damage	5	2 osteoporosis with vertebral fracture,<br>1 osteonecrosis of the hip,<br>1 tendon rupture,<br>1 deforming arthropathy
Gastrointestinal damage	3	1 splenectomy,<br>1 gastric surgery,<br>1 hemicolectomy
Cardiovascular damage	2	1 ventricular dysfunction,<br>1 aortic valvulopathy
Skin damage	1	skin ulceration
premature ovarian failure	1	

Table S4 UCTD Group

Time	Beg. Total	Net Fail	Lost	Nelson-Aalen Cum. Haz.	Std. Error	[95% Conf. Int.]	
2	43	1	0	0.0233	0.0233	0.0033	0.1651
5	42	1	0	0.0471	0.0333	0.0118	0.1882
7	41	1	0	0.0715	0.0413	0.0230	0.2216
8	40	1	0	0.0965	0.0482	0.0362	0.2571
9	39	3	0	0.1734	0.0656	0.0826	0.3639
10	36	1	0	0.2012	0.0712	0.1005	0.4026
11	35	0	30	0.2012	0.0712	0.1005	0.4026
16	5	0	2	0.2012	0.0712	0.1005	0.4026
23	3	0	1	0.2012	0.0712	0.1005	0.4026
25	2	0	1	0.2012	0.0712	0.1005	0.4026
30	1	0	1	0.2012	0.0712	0.1005	0.4026

Table S5 – SLE Group

Time	Beg. Total	Fail	Net Lost	Nelson-Aalen Cum. Haz.	Std. Error	[95% Conf. Int.]	
1	85	4	0	0.0471	0.0235	0.0177	0.1254
2	81	4	0	0.0964	0.0341	0.0482	0.1929
3	77	3	0	0.1354	0.0409	0.0750	0.2446
4	74	3	0	0.1759	0.0471	0.1041	0.2973
5	71	5	0	0.2464	0.0566	0.1570	0.3866
6	66	5	0	0.3221	0.0660	0.2156	0.4813
8	61	3	0	0.3713	0.0719	0.2541	0.5426
9	58	6	0	0.4748	0.0833	0.3365	0.6697
10	52	1	0	0.4940	0.0855	0.3518	0.6936
11	51	0	35	0.4940	0.0855	0.3518	0.6936
12	16	0	3	0.4940	0.0855	0.3518	0.6936
13	13	0	2	0.4940	0.0855	0.3518	0.6936
14	11	0	2	0.4940	0.0855	0.3518	0.6936
15	9	0	2	0.4940	0.0855	0.3518	0.6936
16	7	0	2	0.4940	0.0855	0.3518	0.6936
17	5	0	1	0.4940	0.0855	0.3518	0.6936
18	4	0	2	0.4940	0.0855	0.3518	0.6936
20	2	0	1	0.4940	0.0855	0.3518	0.6936
22	1	0	1	0.4940	0.0855	0.3518	0.6936
